# Coagulation profile evaluated by thromboelastography rotem^®^ in patients with subarachnoid hemorrhage admitted to an intensive care unit

**DOI:** 10.1186/2197-425X-3-S1-A784

**Published:** 2015-10-01

**Authors:** JC Gomez Builes, P Taurà Reverter, E Zavala, A Blasi, M Coca Martinez

**Affiliations:** St Michael's Hospital, Anesthesiology, Toronto, Canada; Hospital Clinic de Barcelona, Anesthesiology and Critical Care, Barcelona, Spain; Hospital Clinic de Barcelona, Anesthesiology, Barcelona, Spain

## Introduction

Coagulation alterations after a subarachnoid hemorrhage (aSAH) secondary to aneurysm rupture are frequent and clinically relevant. They may impact negatively in the outcome. The diagnosis of coagulopathy with the standard tests is limited as only the initial part of coagulation process is being assessed. In contrast, thromboelastography provides the whole blood clot information, allowing evaluation of clot formation, strength and lysis.

## Objective

Describe the coagulation profile in patients with aSAH evaluated by thromboelastography at hospital admission and after 24 hours of symptoms onset.

## Methods

Ethics Board approval and patient consent obtained, this ongoing prospective descriptive study included 8 patients with aSAH admitted to an ICU. Standard coagulation tests (INR, activated partial thromboplastin time, platelet count, Fibrinogen, D- dimer) and thromboelastography ROTEM^®^ were performed at hospital admission and 24 hours after of ictus. Hypocoagulability was defined as FIBTEM^®^ MCF < 9 mm, EXTEM^®^ MCF < 45 mm and G< 5 dynes/cm^2^. Hypercoagulability: EXTEM^®^ MCF > 72 mm or G>11 dynes/cm^2^ and hyperfibrinolysis was defined by ML >17 in EXTEM^®^

## Results

Patients mean (SD) age was 56,6 (10,1) years. At admission all patients had a Fisher Score of 4. Seven patients (87.5%) had D-dimer levels lower than 2500 ng/mL at admission and remained lower in 6 patients (75%) at 24 h. All patients had INR less than 1.3 at admission and normal platelet count. At 24 h one patient (87.5%) had INR >1.3. With ROTEM^®^ analysis hypercoagulability was present in 2 patients (25%) at admission and in 5 patients (62.5%) at 24 h, 2 of them exhibited hyperfibrinolysis. No patients presented hypocoagulability. Clinically, no patients presented rebleeding or vasospasm, 4 patients (50%) had good neurological recovery and 1 patient died in day 6 after hospital admission from a status epilepticus.

## Conclusions

In our study the standard coagulation test did not show significant abnormalities. In contrast ROTEM^®^ tests showed a trend towards a hypercoagulable state as the most frequent coagulation profile. In addition, a trend to increased fibrinolysis was detected. The hemostatic activation caused by aSAH induces an imbalance that may facilitate an increased fibrinolytic activity, compromising the stability and quality of the clot, and may increase the risk of rebleeding.

ROTEM^®^ is useful to detect a real time, hypercoagulable and hyperfibrinolytic activity and could guide the use of antifibrinolytic therapy before the aneurysm treatment or support the clinician in the decision to initiate antithrombotic therapies early in the course of aSAH, after the aneurysm is secured.

**Table 1:** Demographic data and outcome.

**Age years mean (SD)**	56.6 (10.1)
**Female/Male**	5/3
**Hydrocephalus at admission**	6(75%)
**Glasgow Outcome Scale**	
**(5) Good recovery**	4(50%)
**(4) Moderate disability**	2(25%)
**(3) Severe disability**	1(12.5%)
**(2) Persistent vegetative state**	0
**(1) Death**	1(12.5%)

**Table 2:** Coagulation Profiles.

ROTEM^®^ Profile	Admission	24 h
**Hypocoagulability**	0	0
**Normal**	6(75%)	3(37.5%)
**Hypercoagulability**	2(25%)	5 (62.5%)
**Hyperfibrinolysis**	0	2(25%)
**D-Dimer >2500 ng/mL**	1(12.5%)	2(25%)
**INR >1.3**	0	1(12.5%)
**aPTT >35s**	1(12.5%)	0
**Platelets 100 × 109/l (mean)**	188	204
**Fibrinogen g/dL (mean)**	4	3.3
